# SARS-CoV-2 Vaccines and Adverse Effects in Gynecology and Obstetrics: The First Italian Retrospective Study

**DOI:** 10.3390/ijerph192013167

**Published:** 2022-10-13

**Authors:** Miriam Dellino, Bruno Lamanna, Marina Vinciguerra, Silvio Tafuri, Pasquale Stefanizzi, Antonio Malvasi, Giovanni Di Vagno, Gennaro Cormio, Vera Loizzi, Gerardo Cazzato, Raffaele Tinelli, Ettore Cicinelli, Vincenzo Pinto, Antonella Daniele, Eugenio Maiorano, Leonardo Resta, Danila De Vito, Salvatore Scacco, Eliano Cascardi

**Affiliations:** 1 Department of Biomedical Sciences and Human Oncology, Obstetrics and Gynecology Section, Policlinic of Bari, University of Bari “Aldo Moro”, Piazza Aldo Moro, 70100 Bari, Italy; 2 Clinic of Obstetrics and Gynecology, “San Paolo” Hospital, ASL Bari, 70132 Bari, Italy; 3 Fetal Medicine Research Institute, King’s College Hospital, London SE5 9RS, UK; 4 Department of Emergency and Organ Transplantation, Pathology Section, University of Bari “Aldo Moro”, Piazza Giulio Cesare 11, 70124 Bari, Italy; 5 Department of Obstetrics and Gynecology, “Valle d’Itria” Hospital, 74015 Martina Franca, Italy; 6 Experimental Oncology, Center for Study of Heredo-Familial Tumors, IRCCS Istituto Tumori “Giovanni Paolo II” Bari, 70124 Bari, Italy; 7 Department of Basic Medical Sciences and Neurosciences, University of Bari “Aldo Moro”, Piazza Giulio Cesare 11, 70124 Bari, Italy; 8 Department of Medical Sciences, University of Turin, 10124 Turin, Italy; 9 Pathology Unit, FPO-IRCCS Candiolo Cancer Institute, Str. Provinciale 142 km 3.95, 10060 Candiolo, Italy

**Keywords:** SARS-CoV-2, vaccine, gynecological and obstetric effects, anomalous uterine bleeding

## Abstract

The most common effects reported by the Italian Medicine Agency following administration of SARS-CoV-2 vaccine are myalgia, soreness to the arm of inoculation, fever, and asthenia. To date, there are no specific and official reports registered by the Italian Medicine Agency on possible alterations of the menstrual cycle, or of the female reproductive system, following the vaccine. Actually, clinical experience showed a spread of transient adverse drug reactions of the menstrual cycle, following the administration of all COVID-19 vaccine types, both mRNA and Adenovirus vectored ones. In this work, we conducted the first retrospective study on Italian patients vaccinated for SARS-CoV-2 in the period between April 2021 and April 2022, to report the onset of menstrual changes after the vaccine in order to understand: etiology, duration of possible adverse effects, and the extent of the phenomenon. We recruited 100 women aged 18–45, vaccinated for SARS-CoV-2, who were asked to complete a questionnaire consisting of 12 multiple choice questions about the effects of the vaccine on the reproductive system. Thirty-seven of them received three doses of the vaccine, while the remaining 63 received two doses. Symptoms such as delayed menstruation and abnormal uterine bleeding (metrorrhagia, menometrorrhagia, and menorrhagia) were generally reported within the first three weeks of vaccination, especially after the second dose, with a percentage of 23% and 77%, respectively. These preliminary data suggest that this problem may be broader and deserving of further investigation in the future.

## 1. Introduction

The Italian Medicines Agency (AIFA) published data relating to the surveillance of COVID-19 vaccines on the general population from 27 December 2020 to 26 April 2021, from which it emerged that 309 reports have been entered for every 100,000 doses administered, regardless of vaccine type and dose [1]. The vaccines administered to date are: the Pfizer/BioNTech mRNA vaccine called Comirnaty (43%), the Moderna mRNA vaccine called COVID-19 Modern vaccine (32%), and the AstraZeneca recombinant viral vector vaccine, now called Vaxzevria (25%) [2]. Reports mainly concern the Pfizer/BioNTech Comirnaty vaccine (75%), which was the most used (70.9% of the doses administered), and only in a small part the Vaxzevria vaccine (ex COVID-19 Vaccine AstraZeneca; 22%) and the Moderna vaccine (3%) [3]. Furthermore, adverse effects following the vaccine appear numerically higher in a manner directly proportional to the number of doses [1]. For these vaccines administered, adverse events that have been notified and reported by AIFA and the Medicines and Healthcare Products Regulatory Agency (MHRA) are fever, fatigue, headache, muscle/joint pain, injection site pain, chills, and nausea [2]. Investigating all the events that appear after a vaccination serves to collect as much information as possible and to increase the possibility of identifying the suspicious events [3]. It is not always easy to assess whether there is a causal link with vaccination [4]. They could be a symptom of another disease, or they could be associated with another product taken by the person who got vaccinated. Please note that adverse event reports from AIFA represent a snapshot of the reports present in the National Pharmacovigilance Network at the time of data extraction and may change over time [5]. Focusing on the gynecological field, in our clinical practice, shortly after vaccination, transitory period alteration and anomalies vaginal bleeding was reported by a growing number of patients after vaccination [6]. Similarly, from 2 September 2021, these types of reactions have been reported in the literature through all COVID-19 vaccines at MHRA. MHRA had previously analyzed the reports of post-vaccine menstrual disorders, concluding that there was no causal link between vaccines and menstrual cycle alterations. In consideration of further reports on menstrual disorders, the MHRA has decided to investigate the incidence of such cases and to carry out a new analysis of all available data, currently underway [7]. Moreover, these menstrual cycle changes after vaccination seem to return to normal the following cycle. The mechanism for such adverse reactions has not been sufficiently explored. In reality, trials are underway with case control groups (vaccinated and not vaccinated), for which US National Institutes of Health is investing a lot of resources [8]. Pending the results of these ongoing studies, we carried out a survey in the vaccinated population of our outpatient services to evaluate retrospectively, over a year, if the vaccinated patients had reported adverse reactions.

## 2. Materials and Methods

The scientific study is configured as a unicentric and non-profit retrospective investigation. From April 2021 to April 2022 we recruited 100 women, with a middle age of 33 years (range 18–45), who had completed the SARS-CoV-2 vaccination course for 18 months, and who had access to the Gynecology and Obstetrics Unit of the “San Paolo” Hospital for menstrual irregularities and with normal cycle lengths for three consecutive cycles before the first vaccine dose. The average BMI of these women was 27.2. We excluded 259 patients (72%) since they were in menopause, were minors, immune-depressed, pregnant, affected by oncological diseases or by previously recognized gynecological pathologies (fibromatosis or endometrial and ovarian anomalies, polycystic ovary syndrome), in therapy with corticosteroids or contraceptives, or had ongoing vaccines for HPV or other vaccine prophylaxis. Through our data archive, we contacted these patients and asked them to complete a questionnaire consisting of 12 multiple-choice questions (Supplementary Figure S1). Women who agreed to participate to the study signed an informed consent form. All procedures performed in this study were in accordance with the Declaration of Helsinki, as revised in 2013. The clinical data of the patients, together with the results obtained from the questionnaires, were anonymized, reported in a specific database, and subsequently analyzed. Results are presented in percentages.

The data analysis was exploratory, and aimed at describing the information collected in a concise form. The characteristics of the respondents and the responses were summarized by descriptive statistics.

## 3. Results

**Figure 1:**
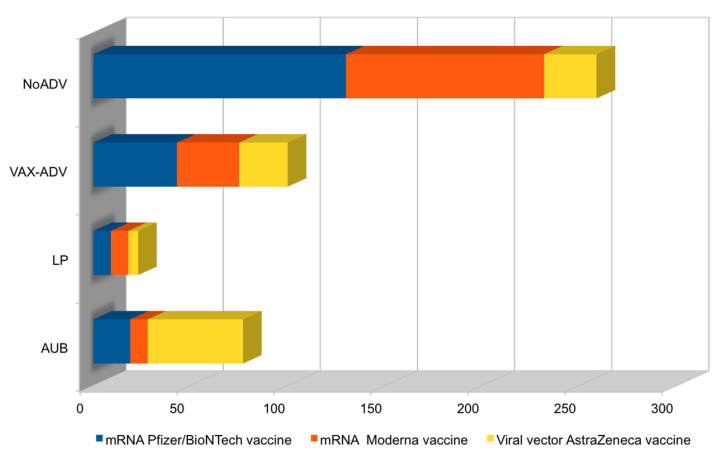
Histogram of COVID-19 vaccine types and corresponding adverse effects. Legend and abbreviations: y = vaccinated women with menstrual irregularities due to other causes, NoADV/vaccinated women whose menstrual irregularities could be adverse effects to vaccine, VAX-ADV/which adverse effect, late period, LP, or abnormal uterine bleeding, AUB; x = number of all vaccinated women with menstrual irregularities.

**Figure 2:**
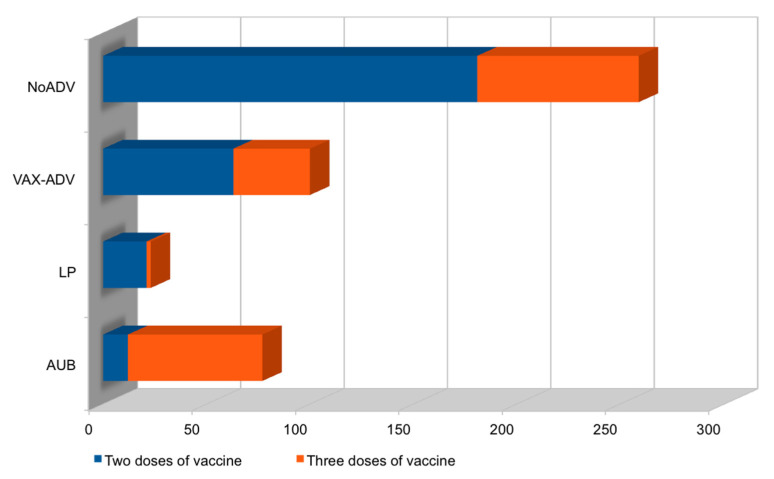
Histogram of COVID-19 vaccines doses and corresponding adverse effects. Legend and abbreviations: y = vaccinated women with menstrual irregularities due to other causes, NoADV/vaccinated women whose menstrual irregularities could be adverse effects to vaccine, VAX-ADV/which adverse effect, late period, LP, or abnormal uterine bleeding, AUB; x = number of all vaccinated women with menstrual irregularities.

We recruited 100 patients aged 18–45 years (average of 33 years), related to outpatient services of the UOC of Gynecology and Obstetrics for menstrual irregularities; they reported having had vaccination for SARS-CoV-2 (with verification of green-pass) and we invited them to complete a questionnaire consisting of 12 questions about the type of menstrual changes they had had and the history of this from the date of the vaccine. From the analysis of these questionnaires, it appears that our patients had received the Pfizer/BioNTech mRNA vaccine (Comirnaty) in 43% of cases, the Moderna mRNA vaccine (COVID-19 Modern Vaccine) in 32% of cases, and the Astrazeneca recombinant viral vector vaccine (Vaxzevria) in 25% of the remaining cases (Figure 1 and Figure 2).

In addition, 37% of them received three doses and 63% received two doses of vaccines. Of all 100 women we recruited (ADV), the average onset of menstrual irregularity was 13 days (1–18) from inoculation of the vaccine. Of these, 90% were after the second dose, and 10% were after the third dose for an average duration of 45.5 days; 15% of the total of these women reported irregularities already after the first dose of vaccine, which reappeared after the second/third dose. In our patient group, 23% had menstrual delay and 77% had abnormal uterine bleeding (AUB), of which 47% had metrorrhagia, 30% had menometrorrhagia, and 23% had menorrhagia (Table 1 and Table 2).

**Figure 3:**
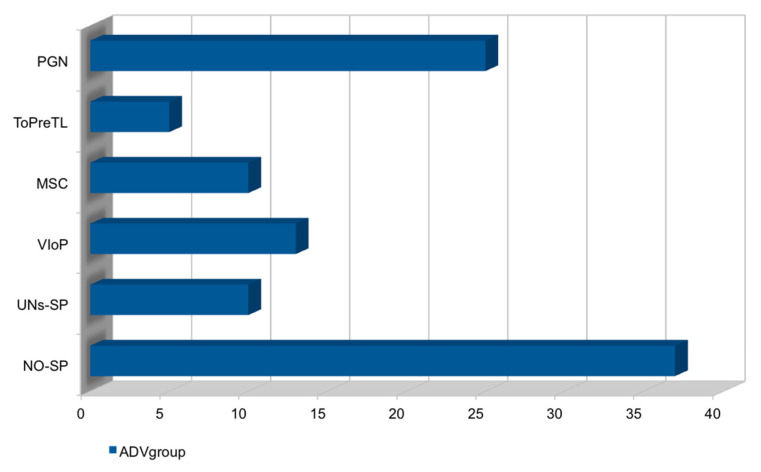
Obstetric outcome in COVID-19 vaccinated women whose menstrual irregularities could be adverse effects to vaccine. Legend and abbreviations: y = women not seeking pregnancy, NO-SP/women unsuccessfully seeking pregnancy, UNs-SP/women undergone voluntary interruption of pregnancy, VIoP/cases of miscarriages, MSC/cases of threat of preterm labor, ToPreTL/women got pregnant, PGN; x = number of vaccinated women.

In cases of AUB, only 28% (27/77) needed therapy with tranexamic acid. In addition, 32% (10/32) of patients contacted the gynecologist/family doctor by short routes without performing a clinical-instrumental assessment, 58% (58/100) had an outpatient specialist examination, and 10% (10/100) had an access to the emergency room (Table 1 and Table 2). Among patients undergoing medical consultations, 38% (12/32) of women reported written documentation. Of these: 57% (7/12) had no ultrasound changes, 30% (3/12) had a fluid effusion layer in Douglas (9 mm average), and 13% (2/12) reported a hemorrhagic corpus luteum. Clinically, 98% (11/12) had abnormal uterine bleeding at the time of the examination. Additionally, 98% (11/12) of patients who reported reactions performed a blood chemistry evaluation with CBC and coagulation with reported mean values of D-DIMERI = 650 (vn: less than 500) ng/mL, PT = 64%, PTT = 31”, Fibrinogen = 331 ng/mL, HB: 11.9 gr/dl, Hematocrit: 37%, PLT: 263 × 1000/uL, WBC: 17.000, 06 × 1000/mL. No one performed iron therapy. In addition, as for the reproductive out-like, after the last dose of vaccine: 25% (25/100) reported to be pregnant, 5% (5/100) reported threat of preterm birth, 10% (10/100) had an abortion, 13% (13/100) had an IVG, 10% (10/100) tried to get pregnant without getting it, and 37% (37/100) did not try to conceive (Figure 3).

Finally, it should also be noted that the total number of accesses for menstrual irregularities between April 2021 and April 2022 was 359 compared to 273 in the period April 2018–April 2019 in the same hospital for the same reasons (+86 patients, 31.5%)

## 4. Discussion

Several questions have arisen about the impact of SARS-CoV-2 vaccination and SARS-CoV-2 infection on future fertility [9,10,11]. Particularly, regarding male fertility, the absence of SARS-CoV-2 in the semen and prostatic secretions of infected patients has been reported in literature [12,13,14,15]; the unlikely possibility of sexual transmission through semen at about 1 month after first detection, patients with a recent infection, or those recovering from COVID-19 has also been reported [13], and SARS-CoV-2 RNA was not detected in semen during the period shortly after infection nor at a later time [14]. There are indirect viral signs, such as testicular injury and inflammatory infiltration, viral orchitis, scrotal discomfort, and altered semen parameters (such as number of spermatozoa with DNA fragmentation). SARS-CoV-2 may lead to infertility through the main receptor binds ACE2 receptor E2 that is widely distributed in the testis, including the Leydig and Sertoli cells [16,17,18,19,20]. Further studies must investigate these aspects and the impact of “long Covid” on male reproduction [21]. On the other hand, even after considering different types of vaccines (mRNA or viral vector), it was reported that COVID-19 vaccination did not damage the sperm quality and fertilization capacity of men (particularly undergoing ART treatments) and should be considered safe for men’s reproductive health [20]. Regarding females, SARS-CoV-2 may invade target ovarian cells by binding to ACE2, altering female fertility [19,22]. ACE2 is widely expressed in the ovaries, uterus, vagina, and placenta, regulating angiotensin II (Ang II) levels to exert its physiological functions, such as follicular development and ovulation, corpus luteum angiogenesis and degeneration, and affect endometrial tissue growth [23,24,25]. Therefore, the ovarian reserve function should be evaluated in order to analyze the impact of COVID-19 on female fertility [23,26,27,28]. On the other hand, low incidence of severe morbidity among pregnancy affected by COVID-19 was described until recent publications have reported severe morbidity and mortality among pregnant affected by the emerging variants of the SARS-CoV-2 virus 9. Consequently, the Center for Disease Control added pregnancy to the list of high-risk conditions to prioritize vaccination and the American College of Obstetricians and Gynecologists recommend vaccination in any stage of the pregnancy [12]. Concerning women eager for offspring, COVID-19 vaccination does not seem to impact fertility, since in clinical trials, adverse pregnancy outcomes occurred with similar rates in vaccinated and unvaccinated groups [21]. Moreover, in assisted reproduction clinics, fertility measures and pregnancy rates are similar in vaccinated and unvaccinated patients [29]. There have been many reports of people with menstrual disturbance following COVID-19 vaccination, including alterations in frequency, duration, regularity, and volume of menstruation [2]. Similarly, in our clinical practice we wondered if we could evaluate the presence of a connection between changes to menstrual periods and COVID-19 vaccines in our population. For this reason, we decided to use a simple questionnaire, which, despite the limits of a retrospective assessment, can represent the attestation of the adverse event and a starting point for further analysis. From the results of the questionnaire, it emerged that symptoms such as delayed menstruation and abnormal uterine bleeding (metrorrhagia, menometrorrhagia, and menorrhagia) were generally reported within the first three weeks of vaccination, especially after the second dose, with a percentage of 23% and 77%, respectively. However, of these, only a limited share had to resort to first-aid access and in none of these cases was an objectionable pathology documented. It can therefore be inferred that the disease triggered by the vaccine was minor and without sequelae. A minority of clinicians (10%) considered it appropriate to treat these alterations and require blood tests. Of these, the interesting result is represented by the finding of D-DIMERI values above the threshold values, which, although similar to the one present in the general population following the vaccine, would deserve further study in our opinion. In addition, in the presence of blood losses, only a limited percentage of patients performed therapy with tranexanic acid, thus strengthening the hypothesis of an occasional and transient phenomenon. Indeed, in the limited works reported in the literature, it has been described that menstrual alteration after COVID-19 vaccination appears to resolve spontaneously and rapidly, which is generally reassuring for subsequent fertility [30]. These studies have recruited a non-uniform sample that includes both individuals that use hormonal contraception and individuals having natural menstrual cycles [31]. Moreover, it is not simple to know if these disturbances are a direct effect of the vaccine itself and the mechanisms causing these effects, as this can change from person to person [31]. Indeed, changes in menstruation may be due to stress, since the female system is designed to momentarily down-regulate to prevent against pregnancy and preserve energy [32]. This mechanism could justify some of the menstrual irregularity detected during the pandemic with COVID-19 or with vaccination [31]. On the other hand, the COVID-19 vaccination originates an immune response and subsequent inflammation may transitorily disturb the ovaric hormonal production over one or two cycles, with consequential anomalous menstrual bleeding [32]. Concerning this hypothesis, a recent study assessed the ovarian involvement in COVID-19 vaccination immune reaction [33]. This research has revealed the presence of anti-SARS-CoV-2 IgG in serum and follicular fluid in recently vaccinated patients versus non-vaccinated non-infected women candidates for vitro fertilization [33]. This research showed that follicular steroidogenesis showed similar and normal rates of estrogen and progesterone production among groups [33]. Moreover, the valuation of the follicular response to the LH/hCG trigger showed a normal and similar response in the different groups [33]. Therefore, despite the evidence of close follicular immune exposure post-infection with SARS-CoV-2 or following BNT162b2 mRNA vaccine, the maturation of the oocyte and its hormonal milieu did not report any measurable modification compared to non-exposed patients [33,34,35].

Our study is a pilot experience which has the limit of being retrospective but can represent a milestone for further study. In particular, the period of observation and the size of the sample should be extended with a multicenter trial in order to provide a greater number of reports and evidence and implement the acquisitions of the AIFA. In fact, for lack of an obvious link of randomness between disorder and COVID-19 vaccine, to date, the number of official reports is low, relative to both the number of people vaccinated and the general prevalence of menstrual disorders [28,36,37]. Only the implementation of this research could lead to real understanding around the mechanism of a hypnotizable association between COVID-19 vaccines and menstrual changes. However, it is important to underline, particularly for women eager for offspring, that by today’s knowledge, these effects in menstrual symptoms do not raise concerns since are transitory, spontaneously resolve, and are much less severe than those associated with COVID-19 infection. Therefore, patients who are called for the vaccine should not be discouraged from obtaining it.

### Limitations of the Study

The work reports the analysis of data collected retrospectively, so this can lend itself to bias due to retrospective collection. It was not possible to consider a control group because in Italy, during the period under review, unvaccinated women could not access the clinic. However, a comparison can be made with the general population. We saw incidences of menstrual irregularities in women of childbearing age (in the absence of organic gynecological pathology, for which an unknown or dysfunctional problem is attributable), in which menstrual irregularities occur in an estimated 14% to 25% of women of childbearing age [38]. We had to deal with a large number of patients with fibroids and climacteric, which are among the main conditions of access to the clinic for menstrual irregularities. Patients by age and BMI were not grouped, because the variability of these factors in the indicated period was almost comparable to that of patients visited in the same hospitals before the pandemic.

## 5. Conclusions

Our research is the first Italian pilot study that investigates and identifies some changes in the menstrual cycle after vaccination with COVID-19 vaccines. Although preliminary, our data, while describing a purely transitory effect of the vaccines examined, represent, in our opinion, an important source of information in order to collect and implement the reports to be submitted to AIFA, both to monitor this phenomenon and for the drafting of new guidelines dedicated to it. Furthermore, from what emerged, the data could have a greater impact at a national and international level. For this reason, our group is already designing more robust research on larger case studies in order to further validate the data that have emerged so far. The future project for this study is to expand it and make it multicentric.

## Figures and Tables

**Table 1:** Adverse effects-group (ADV) characteristics and variables (expressed in percentage, %). Abbreviations: VAX, vaccine; LP, late period; AUB, abnormal uterine bleeding group; dd, days; avg, average.

VAX-AV Onset Window	Symtoms Window	LP	AUB	Menorrhagia	Metrorrhagia	Menometrorrhagia
13 dd avg (1–18)	45 dd avg	23% (23/100) of ADV	77% (77/100) of ADV	23% (18/77) of AUB	47% (36/77) of AUB	30% (23/77) of AUB

**Table 2:** Adverse effects-group (ADV) clinic management tools (expressed in percentage, %). Abbreviations: AUB, abnormal uterine bleeding group.

Tranexamic Acid Use	Physician Consult	Instrumental Exams	Emergency Room Access	Blood Exams
28% (22/77) of AUB	32% (32/100) of ADV	58% (58/100) of ADV	10% (10/100) of ADV	10% (10/100) of ADV

## Data Availability

All results are reported within the text.

## Supplementary Materials

The following supporting information can be downloaded at: https://www.mdpi.com/article/10.3390/ijerph192013167/s1, Figure S1: Questionnaire administered to patients during the investigative phase of the study.
