# Supplementary material for: SARS-CoV-2 Vaccines and Adverse Effects in Gynecology and Obstetrics: The First Italian Retrospective Study
Source: Int J Environ Res Public Health. 2022 Oct 13;19(20):13167. doi: 10.3390/ijerph192013167 (PMC9603573; doi:10.3390/ijerph192013167)
Supplement: Supplementary file 1 [file ijerph-19-13167-s001.zip › ijerph-1923910-supplementary.pdf]

QUESTIONNAIRE FOR PATIENTS VACCINATED FOR COVID-19

(The information you provide will be used anonymously)

AGE: ____________________________________

WEIGHT: ____________________________________

HEIGHT: ____________________________________

CORTICOSTEROID THERAPIES: ____________________________________

OTHER DRUGS: ____________________________________

MESTRUAL REGULARITY: ____________________________________

PARITY: ____________________________________

PREGNANT OR BREASTFEEDING: ____________________________________

PREVIOUS ONCOLOGICAL OR GYNECOLOGICAL PATHOOGIES: ____________________________________

PREVIOUS BLOOD DISORDERS: ____________________________________

OTHER: ____________________________________

1) WHICH VACCINE FOR COVID-19 PERFORMED:

1. Pfizer/BioNTech mRNA vaccine
2. Moderna mRNA vaccine
3. AstraZeneca's recombinant viral vector vaccine

Specify____________________________________

2) HOW MANY DOSES:

1. 1 dose
2. 2 doses
3. 3 doses

Specify____________________________________

3) DO YOU REMEMBER HAVING GYNECOLOGICAL DISORDERS AFTER THE VACCINE?

1. Yes
2. No

Specify____________________________________

4) AFTER WHAT DOSE?

1. 1st dose
2. 2nd dose
3. 3rd dose

Specify____________________________________

5) HOW LONG AFTER ADMINISTRATION OF THE DOSE?

1. One month
2. More than one month
3. Less than one month

Specify____________________________________

6) WHAT KIND OF GYNECOLOGICAL DISORDERS DO YOU REMEMBER HAVING HAD?

1. Menstrual delay
2. Blood losses
3. Other__________________________________

7) HOW LONG DID THEY LAST?

1. One month
2. More than one month
3. Less than one month

Specify____________________________________

8) AS A RESULT OF THESE DISORDERS, HAVE YOU CARRIED OUT ANY INVESTIGATIONS?

1. Outpatient specialist visit
2. Access to the emergency room
3. Consulted the family doctor/gynecologist for short ways
4. Nothing

Specify____________________________________

9) HAVE THE DISORDERS BEEN REPORTED TO A HEALTHCARE PROFESSIONAL?

1. YES
2. NO

Specify____________________________________

10) YOU HAVE DONE:

1. Blood tests
2. Ultrasaund
3. Medications
4. Supplements

Specify____________________________________

11) DO YOU HAVE A REPORTOF THE EXAMS CARRIED OUT?

1. YES
2. NO

Specify____________________________________

12) AFTTER VACCINATION YOU HAD:

1. Pregnancy
2. Miscarriage
3. Birth

Specify____________________________________
